# Effective Targeting of Raf-1 and Its Associated Autophagy by Novel Extracted Peptide for Treating Breast Cancer Cells

**DOI:** 10.3389/fonc.2021.682596

**Published:** 2021-08-27

**Authors:** Hebatullah M. Abou El-Fadl, Naglaa M. Hagag, Reham A. El-Shafei, Mohamed H. Khayri, Gamalat El-Gedawy, Ahmed I. Abd El Maksoud, Doaa D. Mohamed, Dalia D. Mohamed, Ibrahim El Halfawy, Ahmed I. Khoder, Khaled A. Elawdan, Mohamed F. Elshal, Ahmed Salah, Hany Khalil

**Affiliations:** ^1^Genome Department, Animal Health Research Institute, Cairo, Egypt; ^2^Pharmacology Department, Faculty of Veterinary Medicine, Mansoura University, Mansoura, Egypt; ^3^Pharmacology Department, Faculty of Veterinary Medicine, Zagazig University, Zagazig, Egypt; ^4^Department of Clinical Biochemistry and Molecular Diagnostics, National Liver Institute, Menofyia University, Shebin El-Kom, Egypt; ^5^Industrial Biotechnology Department, Genetic Engineering and Biotechnology Research Institute, University of Sadat City, Sadat City, Egypt; ^6^Department of Molecular Diagnostics, Genetic Engineering and Biotechnology Research Institute, University of Sadat City, Sadat City, Egypt; ^7^Department of Molecular Biology, Genetic Engineering and Biotechnology Research Institute, University of Sadat City, Sadat City, Egypt

**Keywords:** breast cancer, Raf-1 activation, autophagy, Sorafenib, honey peptide

## Abstract

Breast cancer is one of the most common causes of death in women worldwide and has harmful influence on their psychological state during therapy. Multikinase inhibitors have become effective drugs for treating a variety of cancer diseases such as breast cancer. A purified short peptide (H-P) was isolated from the natural honey and tested for its potential regulatory role in breast cancer cells compared with the effectiveness of the anticancer drug, Sorafenib (SOR), using MCF-7, EFM-19, and MCF-10A cell lines. Furthermore, we investigated the direct connection between Raf-1 activation and cellular autophagy as potential targets of SOR and H-P extract using RNA interference. Interestingly, the treatment with H-P showed competitive regulation of phosphorylated Raf-1, MEK1/2, and matched autophagy-related LC3B without any detectable toxic effects in the non-tumorigenic epithelial cells. Unlike SOR, the regulation of Raf-1 protein and autophagic machinery by the novel H-P extract showed neglected levels of the released proinflammatory cytokine. This regulation of cytokine secretion by H-P resulted in decreasing the expression level of the transcription factor nuclear factor kappa-light-chain-enhancer of activated B cells (NF-kB) in treated cells. Moreover, the transfection of MCF-7 cells with small interference RNA (siRNA) antagonist Raf-1 expression markedly reduced the expression of LC3B, while it increased the expression of NF-kB1 and NF-kB2, indicating the potential cross-link between Raf-1, autophagy, and NF-kB effector. Collectively, these findings suggest that H-P-mediated Raf-1, MEK1/2, LC3B, and NF-kB provide a novel and efficacious multikinase inhibitor for treating breast cancer without detectable cytotoxic effects.

## Introduction

Breast cancer is a type of cancer that initiates in the breast due to the uncontrolled growth of the cells (1). The type of breast cancer that developed in the breast lobules (lobular carcinoma in situ) or ducts (ductal carcinoma in situ) without immigration is called non-invasive breast cancer, while in invasive breast cancer, the cells can transfer into the nearest breast tissue (2). Noteworthy, the multikinase inhibitors such as Sorafenib (SOR) and Lenvatinib are highly recommended in treating the advanced stage of cancer including lung, liver, and breast cancer (3). SOR is an oral small molecule and one of the tyrosine kinase inhibitors (TKIs) that interrupt many cellular factors including vascular endothelial growth factor (VEGF) and platelet-derived growth factor (PDGF) receptor tyrosine kinases. Moreover, SOR, as a Raf-1 inhibitor, can disturb the RAS/RAF/MEK/ERK signaling pathway in renal cell carcinoma (4–6). Although SOR is efficient for the survival of patients with hepatocellular carcinoma, the increase in some adverse events limits its use in some patients. These adverse events include diarrhea and hand–foot syndrome that have a potent impact on patient life and, in some circumstances, are severe enough to discontinue the use of the drug (7). In addition to the primary resistance, several studies have reported various mechanisms underlying the acquired resistance to SOR, such as the interference between the PI3K/Akt and JAK-STAT pathways, the activation of hypoxia-inducible pathways, and epithelial–mesenchymal transition (8). In breast cancer, SOR has also been studied as a single agent or in combination with other agents. Although in clinical trials, SOR showed limited efficiency in breast cancer, its combination with other drugs has shown potential benefits in progression-free survival (9). In general, several cytotoxic conditions and side effects of the TKIs inhibitors such as SOR have been reported including hypertension, loss of appetite, fatigue, stomach pain, and inflammatory events (10). These inflammatory events are due to the production of proinflammatory cytokines such as tumor necrosis factor-alpha (TNF-α) and interleukin 6 (IL-6), which stimulate the expression of nuclear factor kappa-light-chain-enhancer of activated B cells (NF-kB) (11). Importantly, the most common signal that is regulated by the multikinase inhibitors is the mitogen-activated protein kinase (MAPK) signal transduction, which includes the RAS/RAF/MEK/ERK pathway (12). The key mediator of this pathway is Raf-1, which is responsible for the activation of the subsequent downstream targets such as MEK1 and the transcription factor ERK1/2 (13). Importantly, recent evidence indicated the potential regulatory effect of the RAF/MEK/ERK pathway in controlling the expression levels of autophagy related-8 (Atg8) (14). Mainly, autophagy is an intracellular lysosomal-dependent catabolic process including initiation, elongation, and maturation of specific autophagy vacuoles that engulf cellular components for recycling *via* lysosomal digestion (15, 16). Importantly, the recruiting of autophagy-related proteins family (Atg5-Atg12/Atg16) is required for initiating autophagy, whereas the conversion of unlipidated Atg8, the microtubule-associated protein light chain-3B (LC3B), to lipidated LC3B is essential for the elongation and maturation step (16). Raising evidence indicated a direct association of autophagy in maintaining the solid tumors, in which the cells are in urgent need to resist harmful changes during cell division such as lacking nutrients and oxidative stress (hypoxia) (17, 18). On the other hand, several studies reported the anticancer activity of the naturally purified glycoproteins such as the short peptides extracted from *Achatina fulica* mucus in treating breast cancer (19, 20). Natural honey is rich with such glycoproteins that can be easily purified and tested for treating cancer cells. Based on this, we isolated a short peptide (H-P) from natural honey and tested its biological activity in RAF/MEK/ERK pathway and its associated autophagy as crucial signaling in cancer proliferation using breast cancer cell lines EFM-19 and MCF-7 cells.

## Materials and Methods

### Cell Lines

The metastasis adenocarcinoma MCF-7 cell line (ATCC HTB-22), the invasive ductal carcinoma EFM-19 cell (ACC 231), and the human mammary epithelial MCF-10A cell line (ATCC CRL-10317) were provided by VACSERA (Giza, Egypt). All cell lines were cultured in Roswell Park Memorial Institute (RPMI) medium, which were supplied with 4 mM sodium pyruvate, 4 mM L-glutamine, 100 U/ml penicillin/streptomycin, and 5% bovine serum albumin (BSA) (21). The cells were maintained at 37°C in a humidified 5% CO_2_ incubator (Vision Scientific, Daejeon, South Korea) and were tested for mycoplasma contamination using the cell culture contamination detection kit (Invitrogen, USA).

### Fractionation of the Natural Honey

Natural bee honey was home collected from variable plant sources including clover and lemon flowers. Honey peptide was fractionated and purified under acidic conditions using high-performance liquid chromatography (HPLC, Thermo Fisher Scientific, Madison, USA) as described previously (22). Briefly, 50 g of natural honey was resuspended in 50 ml phosphate buffer saline (PBS) and was subjected to HPLC. The served fraction was further purified to isolate fractions with different peptides based on their molecular weight. The fraction with the molecular weight of 2 kDa or less was collected, and the final concentration was measured by a spectrophotometer. The following amino acid sequence has been identified in the fractionated honey-peptide (H-P): Val-Lys-Gly-Arg-Glu-Asp (patent application 1015/2016, Egypt).

### Cell Viability and Cytotoxicity Assay

The potential cytotoxic induction of both SOR and H-P extract in treated cells was achieved *via* monitoring the cell morphology and accounting the number of survived cells upon treatment. The 50% cytotoxic concentration (CC_50_) of each effector and cell viability rate of treated cells were assessed using WST-1 assay kit (Abcam, USA). Briefly, the same volume of WST-1 reagent was added to 100 µl fluid media of the treated cells in the enzyme-linked immunosorbent assay (ELISA) reader plate and was incubated for 2 h away from the light, and then, the formazan product was measured at the wavelength of 440 nm using an ELISA plate reader (IndiaMART, Delhi, India). Assessing released LDH within the fluid medium of the transfected cells was monitored in a 96-well plate using the LDH production kit (Abcam, ab102526). Following the manufacturing procedures, 100 µl of each sample was incubated with 40 µl LDH buffer and 20 µl substrate for 1 h. Then, the relative production of LDH was calculated by dividing the mean absorbance values of treated cells by the mean absorbance values of the mock, which was indicated by the fold change (23).

### Chemical Treatment

To investigate the effectiveness of the purified H-P and SOR in breast cancer cell proliferation, the MCF-10A, MCF-7, and EFM-19 cells were cultured in a six-well plate with a density of 100,000 cells/well and were exposed to 800 µg/ml of SOR or H-P followed by overnight incubation.

### Transfection Protocol

MCF-7 cells were seeded in a 25-cm cell culture flask with a density of 2 × 10^5^ cells/well and were incubated overnight in the CO_2_ incubator. The transfection of MCF-7 cells, in serum-free medium, with the small interference RNAs (siRNAs) was done by using the HyperFect (Qiagen, USA) following the manufacturer’s instructions. Accordingly, 20 µl HyperFect was gently mixed with 200 ng of the respective siRNA against the coding region of autophagy-related 12 (Atg12), transcript variant 5 (5′-CUUGCUACAUGAAAUGGAUU-3′), or against the coding region of Raf-1, transcript variant 1 (5′-GACAAGCAACACUAUCCGUG-3), or against the Luciferase (5′-AACUUACGCUGAGUACUUCGA-3′), which served as control. The reduced serum media (Opti-MEM, Gibco™, USA) was used to suspend the transfection reagents that were added to MCF-7 for 6 h; then, the transfected cells were incubated with the complete RPMI media for 4 days (24).

### Quantitative Real-Time PCR

The quantitative analysis of mRNA was achieved by quantitative real-time PCR (qRT-PCR) using VeritiPro Thermal Cyclers, 96 wells (Applied Biosystems, Foster, CA). The total RNA was isolated using the TriZol–Phenol–Chloroform (Invitrogen, USA) protocol and was purified using the RNeasy Mini Kit (Qiagen, USA). The quantitative mRNA of Raf-1, MEK1, LC3B, Atg12, NF-kB1, and NF-kB2 was assessed using QuantiTect-SYBR-Green PCR Kit (Qiagen, USA), and the oligonucleotides are listed in **Supplementary Table S1**. The quantification of the housekeeping glyceraldehyde 3-phosphate (GAPDH) mRNA was used for normalization (25, 26). The following reagents have been prepared for each reaction: 10 µl SYBR green, 0.25 µl RNase inhibitor (25 U/µl), 0.5 µl reverse transcriptase (50 U/µl), 1 µl total RNA (100 ng), and 1 µl from each primer up to a final volume of 25 µl using RNase-free water. The following thermal cycling conditions were applied: 50°C for 45 min, 94°C for 5 min, 35 cycles (94°C for 30 s, 58°C for 15 s, 72°C for 30 s).

### ELISA

ELISA was used for the quantification analysis of released interleukin (IL)-6, IL-8, TNF-α, and transforming growth factor-beta (TGF-β) using human ELISA kits (Abcam 46042, Abcam 214030, Abcam 181421, and Abcam 100647, respectively). MCF-7 cells were overnight cultured in a 96-well plate with a density of 10,000 cells/well using the complete RPMI medium. Then, the cells were treated with 800 µg/ml of SOR or H-P followed by the incubation time points of 0, 6, 12, 24, 48, and 72 h. For siRNA transfection, the MCF-7 cells were cultured in a 96-well plate as described above, and each well was transfected with 20 µl Opti-MEM that contains 10 ng of siRNA suspended in 2 µl HyperFect. The transfected cells were incubated for 6 h; then, a fresh RPMI complete medium was added to each well instead of the transfection medium, and the cells were incubated for 1, 2, 3, or 4 days. At each time point, 100 µl of the lysed cells was transferred into the ELISA plate reader and incubated for 3 h at room temperature (RT) with the same volume of the control solution and 50 µl 1× biotinylated antibody. After washing, 100 µl of 1× streptavidin–horseradish peroxidase (HRP) solution was added to the wells, which then were incubated for 30 min away from the light. Finally, 100 µl of the chromogen TMB substrate solution was added to each well followed by a short incubation period for 15 min at RT in the dark. To stop the reaction, 100 µl stop solution was added to each well, and the absorbance of each well was monitored using 450 nm (27).

### Flow Cytometry Analysis

Flow cytometry assay was used to monitor the kinetic protein expression of total Raf-1, phosphorylated-Raf-1 (phospho-Raf-1), phospho-MEK, and lipidated LC3B in MCF-7 cells. Thus, the treated and the transfected cells were washed by PBS and were trypsinized for 3 min. The complete RPMI medium was added to the trypsinized cells, which then were centrifuged for 5 min at 3,000 rpm. The supernatant was removed, and the pellet was resuspended in PBS for washing and was resuspended in PBS with 2% formaldehyde for fixation. For permeabilization, the cells were resuspended in PBS containing Triton X-100 (0.1%) for 3 min. For primary antibody staining, the cells were resuspended and incubated for 2 h at RT in the PBS supplemented with 1% BSA and the diluted rabbit polyclonal anti-LC3BII (1–50) (2775, Cell Signaling Technology, USA). After washing, the cells were centrifuged and resuspended in the PBS that contains 1% BSA and 1–1,000 secondary antibody (goat antirabbit IgG, Alexa Fluor 488, Ab 150129, Abcam, USA). The cells were then incubated away from the light for 2 h. The same conditions were repeated for staining the cells with the primary and secondary antibodies against the total Raf-1 using rabbit polyclonal anti-Raf-1 (1–100) (Ab 137435, Abcam USA) and goat antirabbit IgG (1–1,000) (Alexa Fluor 594, Ab 150092, Abcam, USA) (25). The rabbit monoclonal anti-Raf-1 [phospho S259, EPR3433 (2)] primary (Ab 173539, Abcam, USA) was used to achieve the phospho-Raf-1, and rabbit polyclonal anti-Mek1 + Mek2 (phospho S222) (Ab 4750, Abcam, USA) was used to detect the kinetic expression of phospho-MEK1/2. Finally, the flow cytometry assay (BD Accuri™ C6 Plus Flow Cytometer, BD Bioscience, MI, USA) was used to assess the protein levels using resuspended cells in 500 µl PBS.

### Immunoblotting Analysis

The expression profile of total Raf-1, phospho-Raf-1, Mek1/2, NF-kB, and LCB at the protein level was double checked using immunoblotting analysis. Total protein was extracted from the treated MCF-7 cells using the radioimmunoprecipitation assay (RIPA) lysis and extraction buffer (Thermo Fisher, USA). The extracted proteins were denatured using a loading buffer with the anionic detergent sodium dodecyl sulfate (SDS) and boiled at 95–100°C for 5 min. An equal amount of denatured proteins (100 ng) was loaded in 15% SDS-polyacrylamide gel electrophoresis (PAGE); then, protein electrophoresis was carried out using the Mini-Protean 2-D electrophoresis unit (Bio-Rad, Madison, USA) for 3 h at 100 V. The electrophoresed proteins were then transferred onto nitrocellulose membranes (Millipore, MA, USA) using Bio-Rad electroblotting system (Bio-Rad Mini Trans-Blot Electrophoretic Transfer Cell, Madison, USA) for 3 h at 250 mA at 4°C. The membranes were blocked with 30 ml of 5% non-fat dry milk in Tris-buffered saline with Tween-20 (0.05M Tris–HCl, 0.15M NaCl, 0.1% Tween 20, pH 7.5). Then, the membranes were individually incubated overnight at 4°C with rabbit polyclonal anti-LC3B (1–500), rabbit polyclonal anti-Raf-1 (1–500), rabbit monoclonal anti-Raf-1 (phospho S259) (1–500), rabbit monoclonal anti-Mek1/2 (47E6, mAb) (1–1,000), or rabbit polyclonal anticonjugated NF-kB–P65 (D14E12, mAb) (1–1,000) diluted in the blocking buffer. The membranes were then carefully washed using WesternBreeze solution 16× (Invitrogen, USA) followed by 2 h incubation with mouse monoclonal anti-β-actin (Sigma, Hamburg, Germany). Finally, the membranes were washed and incubated for 1 h at room temperature with either antimouse or antirabbit ready-to-use 2° Solution alkaline phosphates (AP) conjugated (Invitrogen, USA). After washing, the chromogenic detection of desired bands was achieved immediately using AP substrate (WesternBreeze, Invitrogen, USA).

### Statistical Analysis and Prediction Tools

The predicted structure of the amino acid backbone and SOR structure have been designed and performed by the free chemical structrer ChemSpider software provided by the Royal Society of Chemistry, Cambridge, UK (28). The Student’s two-tailed t-test was used for statistical analysis considering p-values ≤0.05 as significant and p-values ≤0.01 as highly significant. Delta–delta Ct analysis was used to calculate the fold changes of the quantitative mRNA assessed by qRT-PCR using the equations: (1) delta Ct = Ct for gene − Ct for GAPDH, (2) delta–delta Ct = delta Ct for experimental − delta Ct for control), and (3) expression fold change = (2 −delta–delta ct) (29).

## Results

### The Cytotoxic Effect of SOR can be Prevented Using the Bee Honey Extract, H-P

**Figure 1:**
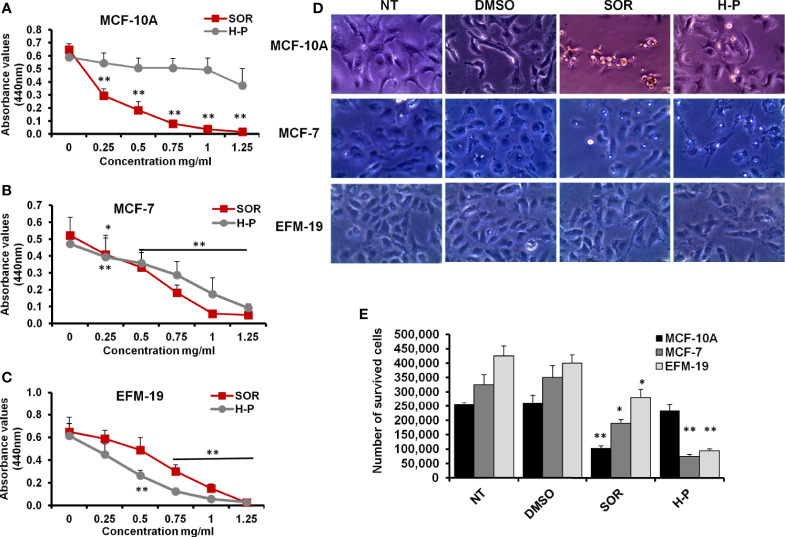
The cytotoxic effects of SOR and H-P extract in normal human mammary cells and breast cancer cells. **(A)** The cell viability rate of MCF-10A cells treated with different concentrations of SOR or H-P extract that indicates the CC_50_ of each one using WST-1 assay, n = 4. **(B)** The cell viability rate of MCF-7 cells treated with different concentrations of SOR or H-P extract that reveals the CC_50_ of each inhibitor using WST-1 assay, n = 4. **(C)** The cell viability rate of EFM-19 cells treated with the same concentrations of SOR or H-P extract and revealed the CC_50_ of each on other breast cancer cells using WST-1 assay, n = 4. Error bars indicate the standard deviation (SD) of four different replicates. **(D)** Representative inverted microscopy images of cells morphology two days upon treatment with SOR or H-P extract in comparison with DMSO-treated cells and untreated cells (NT). **(E)** The number of survived cells treated with either SOR or H-P extract, n = 3. Error bars indicate the SD of three independent experiments. A Student’s two-tailed t-test was used for the significance analysis of represented values. *p ≤ 0.05 and **p ≤ 0.01.

SOR is one of the phenylureas in which one nitrogen is substituted by a 4-chloro-3-trifluorophenyl group, while the other nitrogen is substituted by a phenyl group that is substituted at the para position by a [2-(methylcarbamoyl) pyridin-4-yl] oxy group (**Supplementary Figure S1A**). To investigate the covalent chemical bonds of H-P extract, the peptide backbone based on the binding between the carboxylic group (C1) and the nitrogen number 2 (N2) from the other has been designed by ChemSpider software. As indicated in **Supplementary Figure S1B**, the backbone of the H-P extract consists of Val-Lys-Gly-Arg-Glu-Asp in which the backbone showed several dicarboxylate groups and tagged with aspartic acid. To study the cytotoxic effect of SOR and H-P extract on the breast cancer cells, MCF-7, EFM-19, and the non-tumorigenic epithelial cells (MCF-10A), cells were cultured in 96-well plate and were exposed to a wide concentration range of SOR or H-P effectors (0–1.25 mg/ml). Markedly, both breast cancer cells and normal cell viability rate were significantly interrupted with a lower concentration of SOR (approximately 0.5 mg/ml), while H-P treatment selectively disturbed cancer cell proliferation with an undetectable toxic effect on the MCF-10A cells (**Figures 1A–C** and **Supplementary Tables S2–S4**). Moreover, the cells seeded in a six-well plate were subjected to SOR (800 µg/ml) or H-P extract (800 µg/ml); the concentration inhibited potentially 50% of cell viability (CC_50_), for 2 days, since the mean elimination half-life of SOR is approximately 25–48 h. Notably, an alteration in cell morphology and number of survived cells was observed upon SOR treatment in both cancer and normal cells, while H-P treatment showed significant changes in cancer cell morphology and the number of survived cells without affecting the normal cells (**Figures 1D–F** and **Supplementary Table S5**). These findings indicate that, while SOR caused a potent cytotoxic effect on normal cells (MCF-10A cells), the extracted H-P can prevent cancer cell proliferation without any detectable cytotoxic event in the normal cells.

### Regulation of Raf-1 Activation and Associated Autophagy by SOR and H-P Treatment in MCF-7 Cells

**Figure 2:**
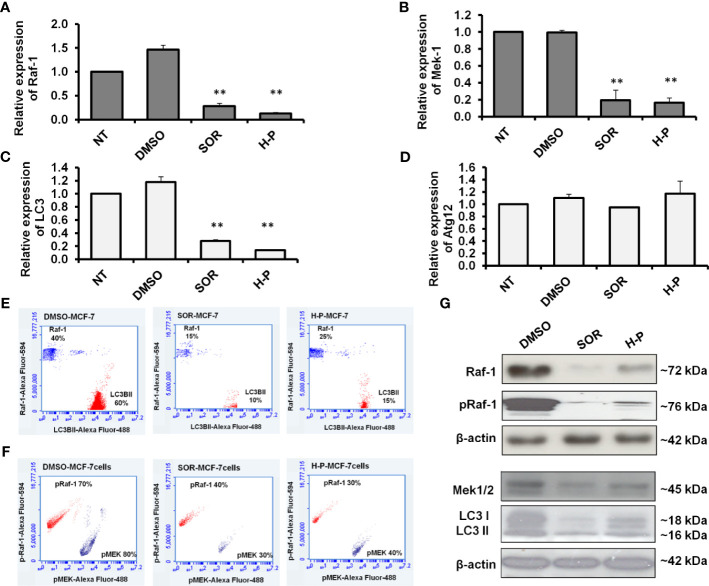
Quantification of Raf-1, MEK1/2, LC3B, and ATG12 expression in SOR and H-P-treated cells. **(A, B)** Steady-state mRNA of Raf-1 and MEK1 indicated by the fold change in MCF-7 cells that were subjected to SOR or H-P extract in comparison with control-treated cells, n = 3. **(C, D)** Steady-state mRNA of autophagy-related LC3B and Atg12 indicated by the fold change in MCF-7 cells treated with SOR or H-P extract in comparison with control-treated cells, n = 3. Levels of GAPDH–mRNA were used as an internal control. Error bars indicate the SD of three independent experiments. **(E)** Quantification analysis of protein profile of both total Raf-1 and lipidated LC3B in treated MCF-7 cells indicated by flow cytometry and compared to DMSO-treated cells. **(F)** Quantification analysis of kinetic protein profile of pRaf-1 and pMEK in MCF-7 cells that were subjected to SOR or H-P indicated by flow cytometry and compared to DMSO-treated cells. **(G)** Immunoblotting investigation of total Raf-1, pRaf-1 Mek1/2, and LC3B proteins in pretreated MCF-7 cells using WesternBreeze Chromogenic Kit. β-Actin served as a loading control. Data are representative of three independent experiments. A Student’s two-tailed t-test was used for significance analysis of cycle threshold (Ct) values. **p ≤ 0.01.

To underline the biological activity of SOR and H-P extract on cellular signaling, we quantified the mRNA of Raf-1, MEK, LC3B, and Atg12 in treated MCF-7 cells using qRT-PCR. Interestingly, the steady-state mRNA of Raf-1 and its downstream target, MEK-1, were significantly reduced in response to SOR and H-P treatment (**Figures 2A, B** and **Supplementary Table S6**). Likewise, the steady-state mRNA of LC3B, but not Atg12, was markedly decreased in both SOR and H-P treated cells in comparison with dimethyl sulfoxide (DMSO)-treated cells (**Figures 2C, D** and **Supplementary Table S6**). Furthermore, MCF-7 cells seeded in a cell culture flask and treated with either SOR or H-P were fixed and stained for the total phospho-Raf-1, phospho-MEK, and the conjugated LC3B protein (LC3BII). Interestingly, SOR and H-P treatment inhibited the constitutive expression of the total Raf-1 and matching LC3BII in the treated cells indicated by the flow cytometry assay. Markedly, the total protein expression of Raf-1 was decreased to approximately 15%; meanwhile, the matched LCBII was decreased to about 10% of stained SOR-treated cells. Likewise, the Raf-1 expression in H-P-treated cells was presented in 25% of the stained cells, while the conjugated LCBII was matched in 15% of the stained cells (**Figure 2E**). In addition, the constitutive activity of both the phospho-Raf-1 and phospho-MEK was decreased to approximately 30% of the stained cells in response to the treatment with either SOR or H-P (**Figure 2F**). Furthermore, the immunoblotting analysis showed that total Raf-1 protein level, phospho-Raf-1, phospho-MEK1/2, and conjugated LC3B were significantly reduced in MCF-7 when treated with either SOR or H-P extract and compared with DMSO-treated cells (**Figure 2G**). These data indicate that H-P extract can regulate the expression of the Raf-1 signaling pathway and its associated autophagic response as a potential synergistic effect in breast cancer cells.

### H-P Mediates the Secretion of Proinflammatory Cytokines Resulted in Low Expression of NF-kB Effectors in Treated Cells

**Figure 3:**
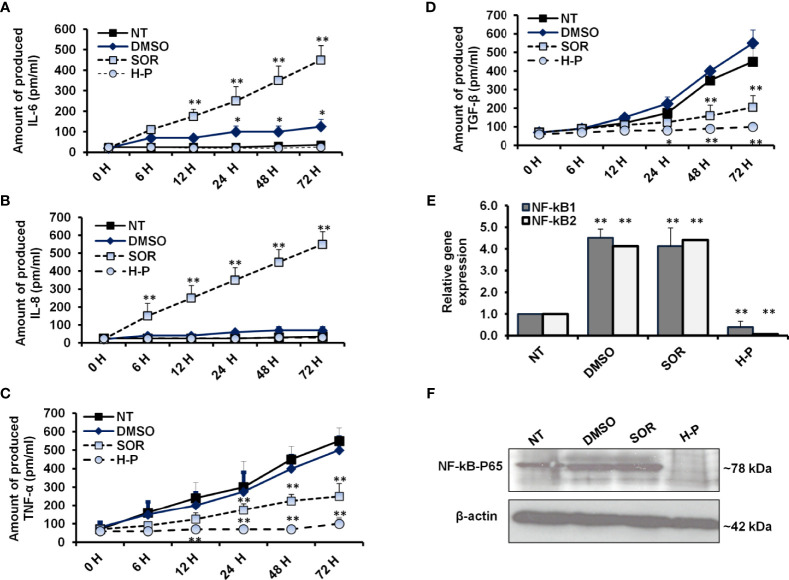
Levels of proinflammatory cytokines and NF-kB effectors in treated-cells. **(A, B)** The concentration of produced IL-6 and IL-8 (pm/ml) in the fluids media of MCF-7 cells that were subjected to 800 µg/ml of either SOR or H-P extract for the indicated time points compared with DMSO-treated cells, n = 4. **(C, D)** Level of produced TNF-α and TGF-β (pm/ml) in the fluid media of MCF-7 cells that were subjected to 800 µg/ml of either SOR or H-P extract for the indicated time points compared with DMSO-treated cells, n = 4. Error bars reveal the SD of four biological replicates. **(E)** Relative gene expression of NF-kB1 and NF-kB2 in treated cells compared with DMSO-treated cells that are quantified by qRT-PCR. Error bars reveal the SD of three independent experiments. **(F)** Immunoblotting analysis of the conjugated NF-kB–P65 in treated MCF-7 cells using WesternBreeze Chromogenic Kit. β-Actin served as a loading control. Data are representative of three independent experiments. A Student’s t-test analysis was performed. *p < 0.05, **p < 0.01.

To measure the released proinflammatory cytokines, we analyzed the fluid medium of the treated MCF-7 cells at the indicated time points using the ELISA test. As shown in **Figure 3A** and **Supplementary Table S7**, the mean concentration of IL-6 was increased to 400 pm/ml in a time-dependent manner of SOR-treated cells, while its concentration was significantly decreased in H-P-treated cells, lower than 100 pm/ml. Likewise, the high level of IL-8 produced in SOR-treated cells was significantly reduced in the cells when treated with the concentration of H-P extract (**Figure 3B** and **Supplementary Table S8**), whereas the mean concentration of TNF-α and TGF-β was significantly decreased in SOR and H-P-treated cells when compared with untreated cells or DMSO-treated cells (**Figures 3C, D** and **Supplementary Tables S9, S10**). Furthermore, the expression of NF-kB effectors as crucial factors regulated by proinflammatory cytokines was achieved in treated cells using qRT-PCR and Western blot. Interestingly, the relative expression of both NF-kB1 and NF-kB2 was significantly downregulated in cells treated with H-P extract, while their expression was significantly increased in cells when treated with SOR (**Figure 3E** and **Supplementary Table S11**). Unlike SOR, the H-P treatment showed a reduced protein level of the conjugated NF-kB–P65 in MCF-7 using immunoblotting assay (**Figure 3F**). These findings demonstrate the ability of H-P extract to adjust the secretion of proinflammatory cytokines and the expression profile of NF-kB effectors in treated cells.

### Knockdown of Raf-1 Correlates With Decreased Autophagy in a Manner Dependent Signaling Intensity

**Figure 4:**
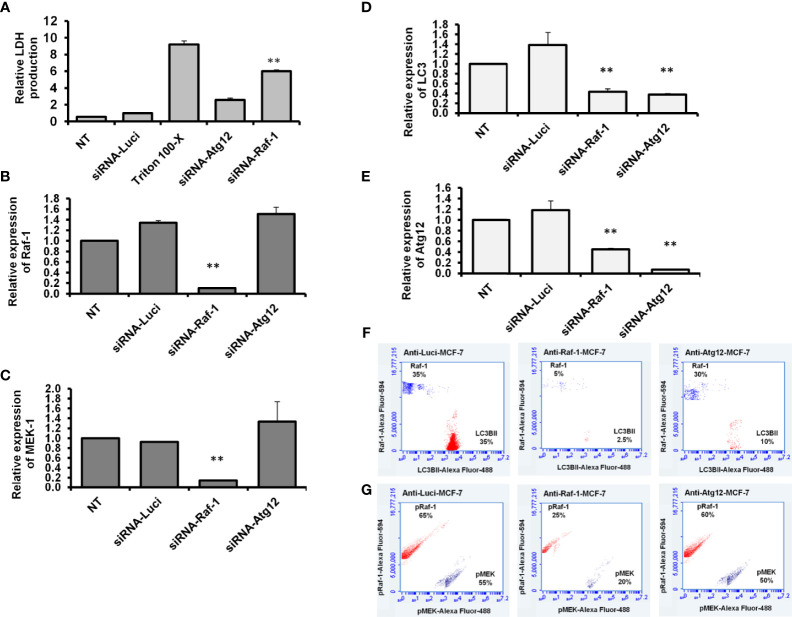
Knockdown efficiency and expression profile of Raf-1, MEK1, LC3B, and ATG12 in siRNA-transfected cells. **(A)** Relative LDH production from transfected cells upon 2 days posttransfection compared with the control-transfected cells, n = 4. Error bars indicate the SD of four biological replicates **(B, C)** Relative gene expression of Raf-1 and MEK1, respectively in siRNA-transfected MCF-7 cells upon 48 h posttransfection compared with the control-transfected cells (siRNA anti-Luciferase). **(D, E)** Relative gene expression of autophagy-related LC3B and Atg12, respectively, indicated by the fold change in siRNA-transfected cells compared with the control-transfected cells. Levels of GAPDH–mRNA were used as an internal control. Error bars indicate the SD of three independent experiments. **(F)** Quantification analysis of total Raf-1 and lipidated LC3B in the transfected MCF-7 cells indicated by flow cytometry and compared with the control-transfected cells. **(G)** Quantification analysis of kinetic protein profile of pRaf-1 and pMEK in transfected MCF-7 cells indicated by flow cytometry and compared to control-transfected cells. Data are representative of three independent experiments. A Student’s t-test analysis was performed. **p < 0.01.

It has been reported that the Raf/MEK/ERK pathway can selectively regulate the expression of autophagy-related LC3B at the mRNA level (14). To confirm the direct interaction between Raf-1 signaling pathway and autophagic machinery, MCF-7 cells were transfected with siRNAs antagonist Raf-1 or Atg12. To determine whether downregulation of Raf-1 or Atg12 affects cell proliferation, LDH production was monitored at 48 h posttransfection. As shown in **Figure 4A**, the transfection with siRNA that targeted Raf-1 significantly increased the production of LDH. In contrast, the transfection with siRNA antagonist Atg12 revealed a comparable level of LDH production when compared with the control-transfected cells. These results indicate that only the downregulation of Raf-1 can disturb cell proliferation and increase LDH production in MCF-7 cells. The knockdown efficiency of Raf-1 revealed a significant reduction of Raf-1–mRNA level in transfected cells with siRNA for Raf-1 knockdown (**Figure 4B** and **Supplementary Table S12**). Furthermore, the relative expression of Raf-1 showed comparable levels in the cells transfected with siRNA for Atg12 knockdown compared with the control-transfected cells (cells transfected with anti-Luciferase siRNA), indicating the negligible effect of Atg12 downregulation on Raf-1 gene expression. Likewise, the quantification analysis of MEK1–mRNA exhibited a significant reduction in cells when transfected with siRNA for Raf-1 knockdown but not in the cells transfected with anti-Atg12 siRNA, indicating the impartial effect of Atg12 knockdown on MEK1 gene expression (**Figure 4C** and **Supplementary Table S12**). By contrast, the quantification analysis of LC3B and Atg12 expression showed significant reduction in mRNA level in transfected cells with either siRNAs antagonist Raf-1 or Atg12, indicating the direct association of autophagy-related genes expression and Raf-1 activity in MCF-7 cells (**Figures 4D, E** **and** **Supplementary Table S12**). In another fashion, the levels of phospho-Raf-1, phospho-MEK, and lipidated LC3B protein were determined in siRNA-transfected cells using flow cytometry assay.

As shown in **Figures 4F, G**, the total Raf-1, phospho-Raf-1, phospho-MEK, and conjugated LC3B protein levels were markedly reduced in cells transfected with siRNA antagonist Raf-1, while their kinetic levels were comparable in MCF-7 cells when transfected with siRNA antagonist Atg12.

Interestingly, matched LC3BII protein was markedly decreased in Raf-1 knockdown cells and Atg12 knockdown cells. These findings suggest that the loss of conjugated LC3BII in MCF-7 cells seems more closely correlated with the loss of Raf-1 activity in cancer cells.

### Raf-1 and Autophagy Tightly Correlated With Proinflammatory Cytokines Sand NF-kB Effectors

**Figure 5:**
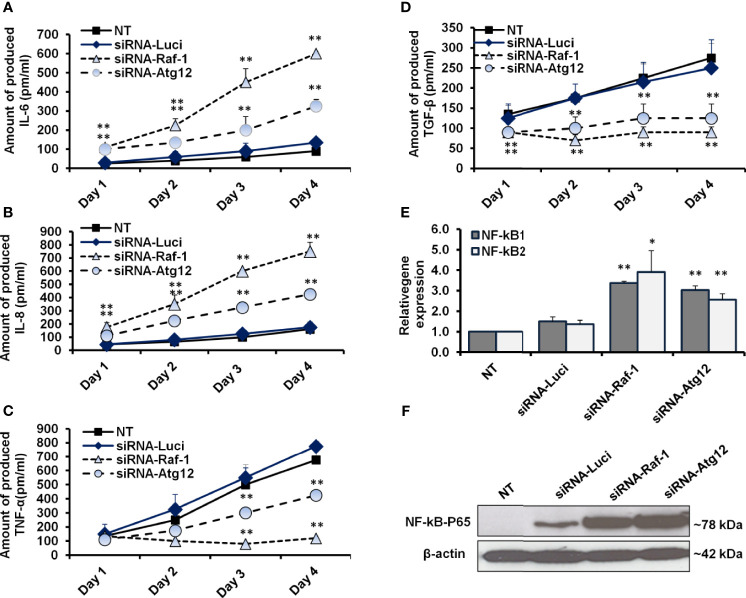
Quantification analysis of proinflammatory cytokines and NF-kB effectors in siRNA-transfected cells. **(A, B)** The concentration of produced IL-6 and IL-8 (pm/ml) in the fluids media of MCF-7 that were transfected with siRNA against Raf-1 or Atg12 compared with the control-transfected cells, n = 4. **(C, D)** Level of produced TNF-α and TGF-β (pm/ml) from MCF-7 cells that were transfected with the indicated siRNAs compared with the control-transfected cells, n = 4. Error bars indicate the SD of four biological replicates. **(E)** Relative gene expression of NF-kB1 and NF-kB2 in transfected cells upon 48 h posttransfection in comparison with control-transfected cells that quantified by qRT-PCR. Error bars indicate the SD of three independent experiments. **(F)** Immunoblotting analysis of the conjugated NF-kB–P65 in transfected MCF-7 cells using WesternBreeze Chromogenic Kit. β-Actin served as a loading control. Data are representative of three independent experiments. A Student’s t-test analysis was performed. *p ≤ 0.05 and **p ≤ 0.01.

To further confirm the correlation between activated Raf-1, autophagy, and production of proinflammatory cytokines, the transfected MCF-7 cells were investigated for their ability to produce IL-6, IL-8, TNF-α, and TGF-β. Interestingly, the ELISA test further revealed a simultaneous increase in IL-6 and IL-8 levels in MCF-7 cells in a time-dependent transfection with siRNAs for either Raf-1 or Atg12 knockdown (**Figures 5A, B** and **Supplementary Tables S13, S14**), while a concurrent reduction in TNF-α and TGF-β was observed in the cells transfected with siRNA antagonist Raf-1 or Atg12 compared with control-transfected cells (**Figures 5C, D** and **Supplementary Tables S15** and **S16**). To investigate whether NF-kB expression is connected with autophagy and Raf-1 activity, the relative expression of NF-kB1 and NF-kB2 was achieved in Raf-1 and Atg12 knockdown cells. Notably, the relative expression of both NF-kB effectors was significantly increased in Raf-1 and Atg12 knockdown cells when compared with control-transfected cells (**Figures 5E** and **Supplementary Tables S17**). Moreover, the immunoblotting analysis of conjugated NF-kB–P65 further confirms the activation of the NF-kB transcription factor in MCF-7 cells associated with Raf-1 and Atg12 downregulation (**Figure 5F**). Together, these observations indicate that downregulation of Raf-1 and Atg12 in MCF-7 cells reduces the production levels of TNF-α and TGF-β while stimulating the production levels of IL-6 and IL-8 that, in turn, stimulates NF-kB effectors in transfected cells.

## Discussion

**Figure 6:**
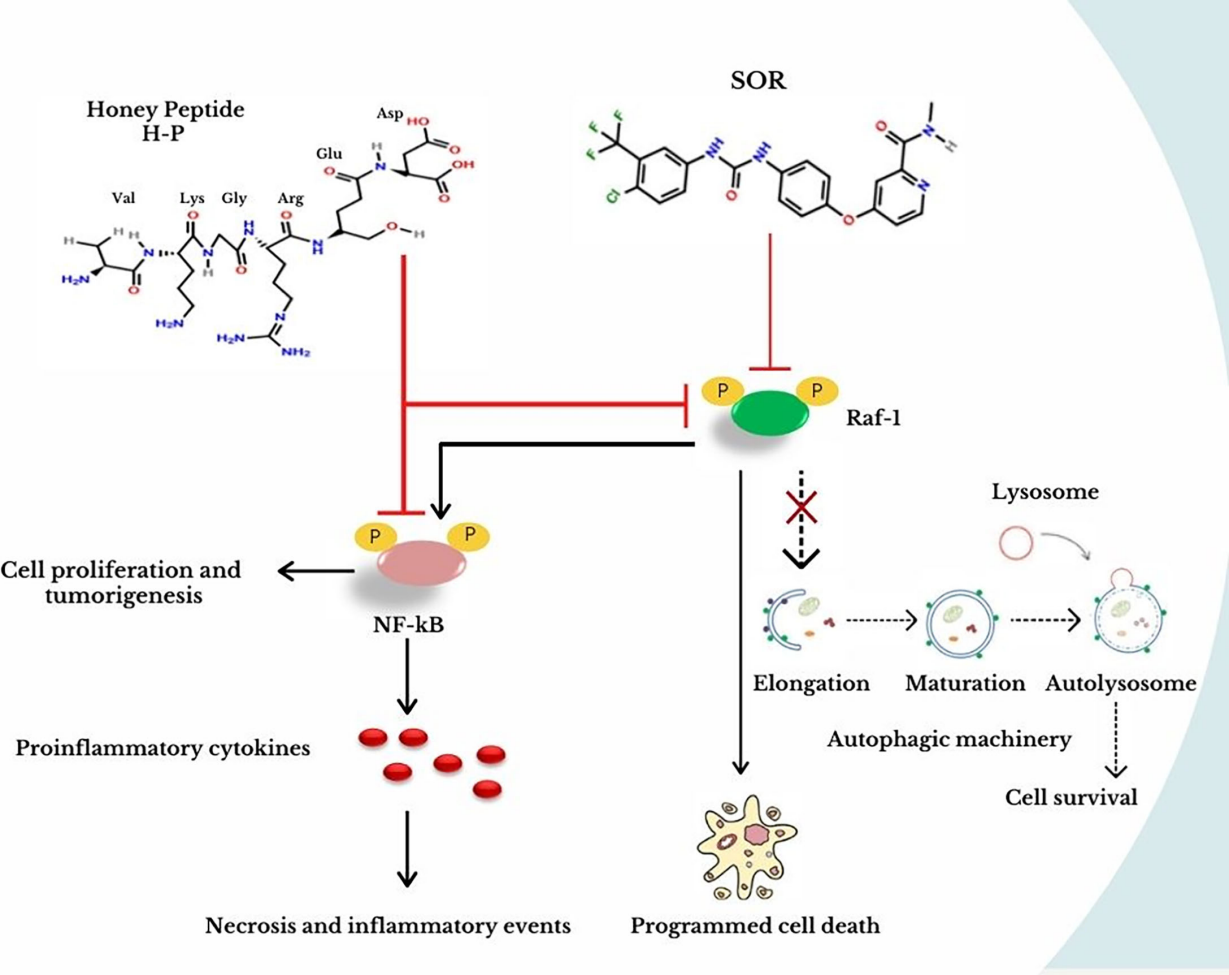
Effective targeting of NF-kB and Raf-1 activation by the novel extracted H-P. In breast cancer cells, SOR treatment inhibits the Raf-1 activity, which prevents the autophagic machinery and stimulates programmed cell death. As a result of this protocol, the secretion of proinflammatory cytokines is increased causing necrotic events and activating NF-kB, which is responsible for cell proliferation and tumorigenesis. In contrast, the treatment of H-P extract can disturb the activity of both Raf-1 and NF-kB, which ensures the best results without detectable cytotoxic effects in normal mammary cells when treating breast cancer.

The present work provides a novel extracted peptide, H-P, which can modulate the Raf-1 signaling pathway, the autophagic machinery, and the expression of NF-kB effectors in breast cancer cells without any cytotoxic effect in the normal mammary cells. Furthermore, the novel extract sufficiently adjusted the production of proinflammatory cytokines including TNF-α, TGF-β, IL-6, and IL-8, which play a crucial role in inflammatory events and cancer development. Mechanistically, the present data indicate that the interrupted signal of the Raf-1/MEK1 pathway is connected with an alteration in lipidated LC3B in a similar pattern that disrupts autophagy. Indeed, the effects of Raf-1 inhibitor, SOR, and Raf-1 knockdown on matching lipidated LC3B further confirm the ability of the Raf/MEK/ERK pathway to alter autophagic activity in breast cancer cells. However, targeting Raf-1 signaling pathway increases the production levels of the proinflammatory cytokines including IL-6 and IL-8 in targeted cells. Noteworthy, the production of IL-6 and IL-8 synergistically activates the expression of NF-kB effectors, which subsequently maintain cell growth and survival, inflammatory events, and cancer development (30, 31). The activation of NF-κB effectors is connected with numerous discrete stimuli such as cytokines, growth factors, mitogens, microbial components, and stress agents (the canonical stimuli), while the non-canonical stimuli of the NF-κB pathway include ligands of a subset of tumor necrosis factor receptor (TNFR) superfamily such as the cluster of differentiation 40 (CD40) and the receptor activator of NF-kB (RANK) (30, 32). Taking this into consideration, we were interested to investigate the ability of the H-P extract to moderate the production of IL-6 and IL-8 and to regulate the Raf-1 signaling pathway in MCF-7 cells compared with SOR. Notably, we selected the MCF-10A cell line as non-tumorigenic epithelial cells to monitor the potential cytotoxic effect of H-P and SOR in the normal epithelial cells. MCF-7 cell line is a commonly used breast cancer cell line that produces essential factors required for angiogenesis and metastasis of breast cancer such as VEGF and TGF due to the expression of estrogen receptor (ER) and progesterone receptor (PR) (33). EFM-19 cell line is a breast ductal carcinoma, which is considered as the most common type of breast cancer with marked expression of both ER and PR (34). Importantly, low levels of IL-6, IL8, TNF-α, and TGF-β were observed in the cells exposed to H-P extract, indicating the ability of H-P to exclude the cytotoxic effect of SOR in treated cells (**Figure 6**). Interestingly, the short peptide drugs have raised attention in cancer therapy, since these peptides can selectively target cancer cells without affecting the normal cells (20, 35). Unlike exploiting a cytotoxic agent, we provide an extracted peptide from the natural honey that prevents the production of proinflammatory cytokines and can be used as an anticancer agent. A very recent study reported the efficiency of certain short-peptide-based chemical groups leading to the prevention of fouling with non-toxic effect and biocompatible coating. These chemical groups contain aspartic acid as an anchoring moiety that exhibited antifouling activity and self-assembling coating. Likewise, the dicarboxylate groups revealed antifouling activity and generate a non-toxic biocompatibility on the desired surface (36). The H-P extract that contains several dicarboxylate groups and tagged with an aspartic acid group showed an obvious regulation of the tumorigenesis NF-kB effector, Raf-1 activity, and conjugated LC3B protein, the key mediator for the maturation of autophagosomes. Noteworthy, SOR has been approved by the US Food and Drug Administration for treating patients with advanced renal carcinoma, hepatocellular carcinoma (HCC), lung cancer, and metastatic breast cancer (37, 38). However, other pathways are underlying the acquired cytotoxic effect and resistance to SOR such as the activation of hypoxia-inducible proteins, JAK-STAT pathway, and PI3K/Akt signaling cascade (8). Nevertheless, the connection between Raf-1 activity and autophagy was first observed *via* detectable alteration in LC3B at mRNA level by the aberrant Raf/MEK/ERK pathway (14). In this way, our findings demonstrate that downregulation of Raf-1 by using respective siRNA reduces lipidated LC3B in transfected MCF-7 cells, while downregulation of Atg12, an essential factor for initiating autophagy, does not modulate Raf-1 expression (**Figure 6**). Thereby, we further confirmed the regulatory role of Raf-1 activation on matching LC3BII at both mRNA and protein levels through targeting of the Raf-1 gene expression in MCF-7 cells. Collectively, the current data provide a novel peptide extracted from the natural honey that can be used as a potential anticancer agent *via* regulating the Raf-1 signaling pathway and avoiding the cytotoxicity and drug resistance to SOR *via* targeting NF-kB effectors. Furthermore, we further investigated the possible connection of Raf-1 phosphorylation and the autophagic process and demonstrated that targeting of Raf-1 signaling cascades can reduce the autophagy-related LCBII protein and certain autophagy machinery. Since it is difficult to extrapolate from the *in vitro* work directly *in vivo*, the current findings need to be confirmed and studied in depth in the xenograft model, in which immunodeficiency mice can be injected with breast cancer cells and treated with the H-P extract to study its effectiveness in preventing the migration and aggregation of the tumor and studying potential side effects.

## Data Availability Statement

The datasets presented in this study can be found in online repositories. The names of the repository/repositories and accession number(s) can be found in the article/**Supplementary Material**.

## Author Contributions

HE-F assisted in performing the experiments. NH assisted in the supervision of the research plan. RE-S and GE-G assisted in performing quantitative real-time PCR experiments. MK helped conceptualize experiments and interpreted data. DaM and DoM assisted in preparing the graphical abstract and chemical models. AM and IH contributed by performing toxicological experiments. ME assisted in providing flow cytometry experiments. AS and AK contributed by performing cell culture experiments and statistical analysis. HK designed the research plan, supervised overall research, provided and interpreted data, and prepared, wrote, and revised the manuscript. All authors contributed to the article and approved the submitted version.

## Funding

The current work was supported within the scope of research projects ID 4694 and 6117 provided by the Egyptian Science and Technology Research Fund (STDF).

## Conflict of Interest

The authors declare that the research was conducted in the absence of any commercial or financial relationships that could be construed as a potential conflict of interest.

## Publisher’s Note

All claims expressed in this article are solely those of the authors and do not necessarily represent those of their affiliated organizations, or those of the publisher, the editors and the reviewers. Any product that may be evaluated in this article, or claim that may be made by its manufacturer, is not guaranteed or endorsed by the publisher.
