# Supplementary material for: Effective Targeting of Raf-1 and Its Associated Autophagy by Novel Extracted Peptide for Treating Breast Cancer Cells
Source: Front Oncol. 2021 Aug 27;11:682596. doi: 10.3389/fonc.2021.682596 (PMC8430328; doi:10.3389/fonc.2021.682596)
Supplement: Supplementary file 1 [file DataSheet_1.doc]

**Table S.1:** Oligonucleotides sequences used for mRNA quantification of indicated genes

| **Description** | **Primer sequences**  **5'-3'** |
| --- | --- |
| **Raf-1-sense** | TTTCCTGGATCATGTTCCCCT |
|  |  |
| **Raf-1 antisense** | ACTTTGGTGCTACAGTGCTCA |
|  |  |
| **MEK1-sense** | GACCTGCGTGCTAGAACCTC |
|  |  |
| **MEK1-antisense** | TCTGGACGCTTGTAGCAGAG |
|  |  |
| **LC3B-sense** | AGAGTCGGATTCGCCGCCGCA |
|  |  |
| **LC3B-antisense** | GACGGCATGGTGCAGGGATCT |
|  |  |
| **Atg12-sense** | CACGAACCATCCAAGGACTCA |
|  |  |
| **Atg12-antisense** | TTTGTGGTTCATCCCCACG |
|  |  |
| **NF-kB1-sense** | GAAATTCCTGATCCAGACAAAAAC |
|  |  |
| **NF-kB1-antisense** | ATCACTTCAATGGCCTCTGTGTAG |
|  |  |
| **NF-kB2-sense** | CTGGTGGACACATACAGGAAGAC |
|  |  |
| **NF-kB2-antisense** | ATAGGCACTGTCTTCTTTCACCTC |
|  |  |
| **GAPDH-sense** | TGGCATTGTGGAAGGGCTCA |
|  |  |
| **GAPDH-antisense** | TGGATGCAGGGATGATGTTCT |

**Figure S.1:** The represented model of the covalent chemical bond for SOR agent **(A)** and H-P extract **(B)** that designed by ChemSpider software.

**
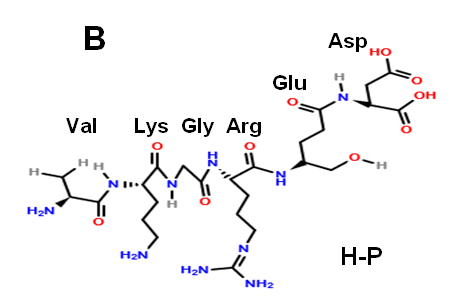
**

**
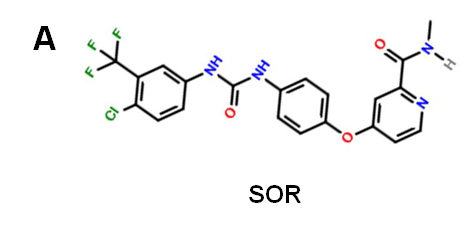
**

**Table S.2:** Statistical analysis of MCF-10A cell viability upon chemical treatment indicated by absorbance values

| **Treatment** | **Concentration**  **(mg/ml)** | **Mean** | **Standard deviation** | **Student two tails t-test** | ***P*-values** |
| --- | --- | --- | --- | --- | --- |
| **SOR**  **H-P** | **0.0^¥^**  **0.25**  **0.5**  **0.75**  **1.0**  **1.25**  **0.0^¥^**  **0.25**  **0.5**  **0.75**  **1.0**  **1.25** | 0.65  0.30  0.15  0.08  0.04  0.02  0.60  0.55  0.50  0.50  0.50  0.45 | 0.05  0.05  0.07  0.02  0.01  0.01  0.01  0.08  0.08  0.07  0.09  0.15 | 0.005  0.003  0.002  0.001  0.001  0.25  0.07  0.07  0.07  0.09 | ≤ 0.01**  ≤ 0.01**  ≤ 0.01**  ≤ 0.01**  ≤ 0.01**    > 0.05  > 0.05  > 0.05  > 0.05  > 0.05 |
|  |  |  |  |  |  |

**^¥^:** DMSO treatment (Control)

**: Highly significant values

**Table S.3:** Statistical analysis of MCF-7 cell viability upon chemical treatment indicated by absorbance values

| **Treatment** | **Concentration**  **(mg/ml)** | **Mean** | **Standard deviation** | **Student two tails t-test** | ***P*-values** |
| --- | --- | --- | --- | --- | --- |
| **SOR**  **H-P** | **0.0**  **0.25**  **0.5**  **0.75**  **1.0**  **1.25**  **0.0**  **0.25**  **0.5**  **0.75**  **1.0**  **1.25** | 0.52  0.41  0.33  0.18  0.06  0.05  0.47  0.40  0.36  0.29  0.18  0.09 | 0.11  0.10  0.11  0.05  0.02  0.07  0.03  0.13  0.06  0.08  0.10  0.01 | 0.17  0.047  0.001  0.001  0.001  0.28  0.001  0.004  0.001  0.001 | > 0.05  ≤ 0.05*  ≤ 0.01**  ≤ 0.01**  ≤ 0.01**    > 0.05  ≤ 0.01**  ≤ 0.01**  ≤ 0.01**  ≤ 0.01** |
|  |  |  |  |  |  |

*: Significant values

**Table S.4:** Statistical analysis of EFM-19 cell viability upon chemical treatment indicated by absorbance values

| **Treatment** | **Concentration**  **(mg/ml)** | **Mean** | **Standard deviation** | **Student two tails t-test** | ***P*-values** |
| --- | --- | --- | --- | --- | --- |
| **SOR**  **H-P** | **0.0**  **0.25**  **0.5**  **0.75**  **1.0**  **1.25**  **0.0**  **0.25**  **0.5**  **0.75**  **1.0**  **1.25** | 0.65  0.60  0.50  0.30  0.15  0.03  0.60  0.45  0.26  0.12  0.06  0.03 | 0.13  0.08  0.11  0.06  0.04  0.02  0.11  0.09  0.05  0.03  0.01  0.02 | 0.45  0.10  0.002  0.003  0.001  0.06  0.001  0.001  0.001  0.002 | > 0.05  > 0.05  ≤ 0.01**  ≤ 0.01**  ≤ 0.01**  > 0.05  ≤ 0.01**  ≤ 0.01**  ≤ 0.01**  ≤ 0.01** |
|  |  |  |  |  |  |

**Table S.5:** Statistical analysis of number of survived cells upon chemical treatment.

| **Cell line** | **Treatment** | **Average** | **Standard deviation** | **Student two tails t-test** | ***P*-values** |
| --- | --- | --- | --- | --- | --- |
| **MCF-10A**  **MCF-7**  **EFM-19** | **NT**  **DMSO**  **SOR**  **H-P**  **NT**  **DMSO**  **SOR**  **H-P**  **NT**  **DMSO**  **SOR**  **H-P** | 255000  260000  110000  260000  325000  350000  190000  75000  425000  400000  280000  95000 | 7071  28284  7071  21213  35355  42426  14142  7071  35355  28284  28284  7071 | 0.83  0.002  0.42  0.58  0.038  0.01  0.51  0.045  0.005 | > 0.05  ≤ 0.01**  > 0.05  > 0.05  ≤ 0.05*  ≤ 0.01**  > 0.05  ≤ 0.05*  ≤ 0.01** |
|  |  |  |  |  |  |

**NT:** Nontreated cells (Control)

**Table S.6:** Quantification analysis of the relative gene expression of Atg12, LC3B, Raf-1, and MEK1effectors in MCF-7 cells treated with SOR and H-P extract.

| **Genes** | **Conditions** | **Fold changes** | **Standard deviation** | **Student two tails t-test** | ***P*-values** |
| --- | --- | --- | --- | --- | --- |
| **Raf-1**  **MEK1**  **LC3B**  **Atg12** | **SOR**  **H-P**  **SOR**  **H-P**  **SOR**  **H-P**  **SOR**  **H-P** | 0.28  0.13  0.19  0.17  0.28  0.14  0.96  1.17 | 0.06  0.02  0.12  0.06  0.02  0.01  0.01  0.21 | 0.001  0.002  0.001  0.002  0.005  0.001  0.17  0.35 | ≤ 0.01**  ≤ 0.01**  ≤ 0.01**  ≤ 0.01**  ≤ 0.01**  ≤ 0.01**  ˃ 0.05  ˃ 0.05 |
|  |  |  |  |  |  |

**Table S.7:** Statistical analysis of produced IL-6 from treated MCF-7 cells subjected to 800µg/ul anti-cancer inhibitor compared with nontreated cells (NT).

| **Treatment** | **Time**  **table** | **Mean**  **Concentration**  **(pm/ml)** | **Standard deviation** | **Student two tails t-test** | ***P*-values** |
| --- | --- | --- | --- | --- | --- |
| **NT**  **DMSO**  **SOR**  **H-P** | **0 H**  **6 H**  **12 H**  **24 H**  **48 H**  **72 H**  **0 H**  **6 H**  **12 H**  **24 H**  **48 H**  **72 H**  **0 H**  **6 H**  **12 H**  **24 H**  **48 H**  **72 H**  **0 H**  **6 H**  **12 H**  **24 H**  **48 H**  **72 H** | 20  20  20  30  30  30  20  20  30  60  80  100  20  80  180  260  320  400  20  20  20  30  30  30 | 3.5  5.5  5.5  2.5  5.5  5.0  2.5  2.5  5.5  5.5  10  10  2.5  2.5  25.5  25.5  30  30  2.5  2.5  5  5  5.5  5.5 | 0.24  0.28  0.2  0.05  0.03  0.04  0.15  0.25  0.001  0.004  0.001  0.001  0.25  0.30  0.45  0.40  0.25  0.35 | > 0.05  > 0.05  > 0.05  ≤ 0.05*  < 0.05*  < 0.05*    > 0.05  > 0.05  ≤ 0.01**  ≤ 0.01**  ≤ 0.01**  ≤ 0.01**  > 0.05  > 0.05  > 0.05  > 0.05  > 0.05  > 0.05 |
|  |  |  |  |  |  |

**Table S.8:** Statistical analysis of produced IL-8 from treated MCF-7 cells subjected to 800µg/ul anti-cancer inhibitor compared with nontreated cells (NT).

| **Treatment** | **Time**  **table** | **Mean**  **Concentration**  **(pm/ml)** | **Standard deviation** | **Student two tails t-test** | ***P*-values** |
| --- | --- | --- | --- | --- | --- |
| **NT**  **DMSO**  **SOR**  **H-P** | **0 H**  **6 H**  **12 H**  **24 H**  **48 H**  **72 H**  **0 H**  **6 H**  **12 H**  **24 H**  **48 H**  **72 H**  **0 H**  **6 H**  **12 H**  **24 H**  **48 H**  **72 H**  **0 H**  **6 H**  **12 H**  **24 H**  **48 H**  **72 H** | 20  20  20  30  30  30  20  20  30  30  40  40  20  150  250  350  450  550  20  20  20  20  20  30 | 3.5  5.5  5.5  2.5  5.5  5  2.5  2.5  5.5  5.5  5.5  5.5  2.5  7.0  14  7  14  14  2.5  2.5  2  2  2.5  2.5 | 0.24  0.28  0.2  0.25  0.25  0.15  0.15  0.01  0.001  0.004  0.002  0.002  0.20  0.30  0.40  0.20  0.20  0.25 | > 0.05  > 0.05  > 0.05  > 0.05  > 0.05  > 0.05    > 0.05  ≤ 0.01**  ≤ 0.01**  ≤ 0.01**  ≤ 0.01**  ≤ 0.01**  > 0.05  > 0.05  > 0.05  > 0.05  > 0.05  > 0.05 |
|  |  |  |  |  |  |

**Table S.9:** Statistical analysis of produced TNF-α from treated MCF-7 cells subjected to 800µg/ul anti-cancer inhibitor compared with nontreated cells (NT).

| **Treatment** | **Time**  **table** | **Mean**  **Concentration**  **(pm/ml)** | **Standard deviation** | **Student two tails t-test** | ***P*-values** |
| --- | --- | --- | --- | --- | --- |
| **NT**  **DMSO**  **SOR**  **H-P** | **0 H**  **6 H**  **12 H**  **24 H**  **48 H**  **72 H**  **0 H**  **6 H**  **12 H**  **24 H**  **48 H**  **72 H**  **0 H**  **6 H**  **12 H**  **24 H**  **48 H**  **72 H**  **0 H**  **6 H**  **12 H**  **24 H**  **48 H**  **72 H** | 70  160  240  300  450  550  80  150  200  275  400  500  70  90  125  175  225  250  60  70  70  70  70  100 | 14  56  84  141  70  70  28  70  70  106  70  70  14  56  35  35  35  70  0  28  14  14  14  28 | 0.15  0.35  0.25  0.50  0.25  0.15  0.15  0.06  0.001  0.004  0.002  0.002  0.20  0.07  0.001  0.002  0.001  0.01 | > 0.05  > 0.05  > 0.05  > 0.05  > 0.05  > 0.05    > 0.05  > 0.05  ≤ 0.01**  ≤ 0.01**  ≤ 0.01**  ≤ 0.01**  > 0.05  > 0.05  ≤ 0.01**  ≤ 0.01**  ≤ 0.01**  ≤ 0.01** |
|  |  |  |  |  |  |

**Table S.10:** Statistical analysis of produced TGF-β from treated MCF-7 cells subjected to 800µg/ul anti-cancer inhibitor compared with nontreated cells (NT).

| **Treatment** | **Time**  **table** | **Mean**  **Concentration**  **(pm/ml)** | **Standard deviation** | **Student two tails t-test** | ***P*-values** |
| --- | --- | --- | --- | --- | --- |
| **NT**  **DMSO**  **SOR**  **H-P** | **0 H**  **6 H**  **12 H**  **24 H**  **48 H**  **72 H**  **0 H**  **6 H**  **12 H**  **24 H**  **48 H**  **72 H**  **0 H**  **6 H**  **12 H**  **24 H**  **48 H**  **72 H**  **0 H**  **6 H**  **12 H**  **24 H**  **48 H**  **72 H** | 70  90  120  175  350  450  70  90  150  225  400  550  70  90  110  125  160  205  60  70  80  80  90  100 | 14  10  0  35  70  70  14  14  0  35  0  70  14  56  35  35  35  70  00  28  35  14  0  0 | 0.25  0.15  0.5  0.5  0.5  0.25  0.15  0.21  0.12  0.15  0.001  0.001  0.20  0.07  0.06  0.05  0.001  0.001 | > 0.05  > 0.05  > 0.05  > 0.05  > 0.05  > 0.05    > 0.05  > 0.05  > 0.05  > 0.05  ≤ 0.01**  ≤ 0.01**  > 0.05  > 0.05  > 0.05  ≤ 0.05*  ≤ 0.01**  ≤ 0.01** |
|  |  |  |  |  |  |

**Table S.11:** Quantification analysis of the relative gene expression of NF-kB1 and NF-kB2 in MCF-7 cells transfected with siRNA against Raf-1 or siRNA against Atg12 compared with control-transfected cells.

| **Genes** | **Treatment** | **Fold changes** | **Standard deviation** | **Student two tails t-test** | ***P*-values** |
| --- | --- | --- | --- | --- | --- |
| **NF-kB1**  **NF-kB2** | **DMSO**  **SOR**  **H-P**  **DMSO**  **SOR**  **H-P** | 4.23  4.73  0.59  4.13  4.41  0.06 | 0.41  0.85  0.28  0.06  1.33  0.06 | 0.001  0.002  0.01  0.001  0.002  0.004 | ≤ 0.01**  ≤ 0.01**  ≤ 0.01**  ≤ 0.01**  ≤ 0.01**  ≤ 0.01** |
|  |  |  |  |  |  |

**Table S.12:** Quantification analysis of the relative gene expression of Atg12, LC3B, Raf-1, and MEK1effectors in MCF-7 cells transfected with siRNA against Raf-1 or siRNA against Atg12 compared with control-transfected cells.

| **Genes** | **Conditions** | **Fold changes** | **Standard deviation** | **Student two tails t-test** | ***P*-values** |
| --- | --- | --- | --- | --- | --- |
| **Raf-1**  **MEK1**  **LC3B**  **Atg12**  **NF-kB1**  **NF-kB2** | **Anti-Luciferase**  **Anti-Raf-1**  **Anti-Atg12**  **Anti-Luciferase**  **Anti-Raf-1**  **Anti-Atg12**  **Anti-Luciferase**  **Anti-Raf-1**  **Anti-Atg12**  **Anti-Luciferase**  **Anti-Raf-1**  **Anti-Atg12**  **Anti-Luciferase**  **Anti-Raf-1**  **Anti-Atg12**  **Anti-Luciferase**  **Anti-Raf-1**  **Anti-Atg12** | 1.34  0.10  1.50  0.92  0.15  1.33  1.38  0.43  0.37  1.18  0.44  0.07  1.5  3.3  3.04  1.3  3.1  2.5 | 0.04  0.01  0.13  0.08  0.01  0.40  0.25  0.06  0.02  0.17  0.02  0.08  0.23  0.09  0.2  0.2  1.04  0.3 | 0.081  0.001  0.032  0.06  0.002  0.40  0.17  0.006  0.009  0.27  0.009  0.008  0.09  0.008  0.005  0.21  0.01  0.001 | ˃ 0.05  ≤ 0.01**  ≤ 0.05*  ˃ 0.05  ≤ 0.01**  ˃ 0.05  ˃ 0.05  ≤ 0.01**  ≤ 0.01**  ˃ 0.05  ≤ 0.01**  ≤ 0.01**  ˃ 0.05  ≤ 0.01**  ˃ 0.01**  ˃ 0.05  ≤ 0.05*  ˃ 0.01** |
|  |  |  |  |  |  |

**Table S.13:** Statistical analysis of produced IL-6 from transfected MCF-7 cells compared with nontransfected cells (NT).

| **Treatment** | **Time**  **table** | **Mean**  **Concentration**  **(pm/ml)** | **Standard deviation** | **Student two tails t-test** | ***P*-values** |
| --- | --- | --- | --- | --- | --- |
| **NT**  **siRNA-Luciferase**  **siRNA-Raf-1**  **siRNA-Atg12** | **Day 1**  **Day 2**  **Day 3**  **Day 4**      **Day 1**  **Day 2**  **Day 3**  **Day 4**  **Day 1**  **Day 2**  **Day 3**  **Day 4**    **Day 1**  **Day 2**  **Day 3**  **Day 4** | 25  40  60  90  30  60  90  135  110  225  450  600  100  135  200  325 | 7  14  28  14  14  28  42  21  14  35  70  35  28  14  14  14 | 0.15  0.35  0.25  0.50  0.01  0.001  0.001  0.002  0.01  0.001  0.002  0.001 | > 0.05  > 0.05  > 0.05  > 0.05      ≤ 0.01**  ≤ 0.01**  ≤ 0.01**  ≤ 0.01**  ≤ 0.01**  ≤ 0.01**  ≤ 0.01**  ≤ 0.01** |
|  |  |  |  |  |  |

**Table S.14:** Statistical analysis of produced IL-8 from transfected MCF-7 cells compared with nontransfected cells (NT).

| **Treatment** | **Time**  **table** | **Mean**  **Concentration**  **(pm/ml)** | **Standard deviation** | **Student two tails t-test** | ***P*-values** |
| --- | --- | --- | --- | --- | --- |
| **NT**  **siRNA-Luciferase**  **siRNA-Raf-1**  **siRNA-Atg12** | **Day 1**  **Day 2**  **Day 3**  **Day 4**      **Day 1**  **Day 2**  **Day 3**  **Day 4**  **Day 1**  **Day 2**  **Day 3**  **Day 4**    **Day 1**  **Day 2**  **Day 3**  **Day 4** | 45  65  100  160  45  80  125  175  175  350  600  750  110  225  325  425 | 7  21  28  56  7  28  35  35  35  70  35  70  14  35  35  35 | 0.5  0.25  0.5  0.55  0.01  0.002  0.004  0.002  0.001  0.003  0.002  0.002 | > 0.05  > 0.05  > 0.05  > 0.05      ≤ 0.01**  ≤ 0.01**  ≤ 0.01**  ≤ 0.01**  ≤ 0.01**  ≤ 0.01**  ≤ 0.01**  ≤ 0.01** |
|  |  |  |  |  |  |

**Table S.15:** Statistical analysis of produced TNF-α from transfected MCF-7 cells compared with nontransfected cells (NT).

| **Treatment** | **Time**  **table** | **Mean**  **Concentration**  **(pm/ml)** | **Standard deviation** | **Student two tails t-test** | ***P*-values** |
| --- | --- | --- | --- | --- | --- |
| **NT**  **siRNA-Luciferase**  **siRNA-Raf-1**  **siRNA-Atg12** | **Day 1**  **Day 2**  **Day 3**  **Day 4**      **Day 1**  **Day 2**  **Day 3**  **Day 4**  **Day 1**  **Day 2**  **Day 3**  **Day 4**    **Day 1**  **Day 2**  **Day 3**  **Day 4** | 135  250  500  650  150  300  550  700  135  100  80  120  110  175  300  425 | 21  70  141  104  70  106  70  35  21  35  0  0  14  56  0  35 | 0.5  0.25  0.5  0.55  0.06  0.07  0.001  0.001  0.06  0.06  0.002  0.002 | > 0.05  > 0.05  > 0.05  > 0.05      > 0.05  > 0.05  ≤ 0.01**  ≤ 0.01**  > 0.05  > 0.05  ≤ 0.01**  ≤ 0.01** |
|  |  |  |  |  |  |

**Table S.16:** Statistical analysis of produced TGF-β from transfected MCF-7 cells compared with nontransfected cells (NT).

| **Treatment** | **Time**  **table** | **Mean**  **Concentration**  **(pm/ml)** | **Standard deviation** | **Student two tails t-test** | ***P*-values** |
| --- | --- | --- | --- | --- | --- |
| **NT**  **siRNA-Luciferase**  **siRNA-Raf-1**  **siRNA-Atg12** | **Day 1**  **Day 2**  **Day 3**  **Day 4**      **Day 1**  **Day 2**  **Day 3**  **Day 4**  **Day 1**  **Day 2**  **Day 3**  **Day 4**    **Day 1**  **Day 2**  **Day 3**  **Day 4** | 135  175  225  275  125  175  215  230  90  70  90  90  90  100  125  125 | 21  35  35  35  35  35  70  70  14  14  14  14  14  28  35  35 | 0.55  0.25  0.15  0.25  0.001  0.002  0.001  0.001  0.001  0.002  0.001  0.001 | > 0.05  > 0.05  > 0.05  > 0.05      ≤ 0.01**  ≤ 0.01**  ≤ 0.01**  ≤ 0.01**  ≤ 0.01**  ≤ 0.01**  ≤ 0.01**  ≤ 0.01** |
|  |  |  |  |  |  |

**Table S.17:** Quantification analysis of the relative gene expression of NF-kB1 and NF-kB2 in MCF-7 cells transfected with siRNA against Raf-1 or siRNA against Atg12 compared with control-transfected cells.

| **Genes** | **Treatment** | **Fold changes** | **Standard deviation** | **Student two tails t-test** | ***P*-values** |
| --- | --- | --- | --- | --- | --- |
| **NF-kB1**  **NF-kB2** | **Anti-Luci**  **Anti-Raf-1**  **Anti-Atg12**  **Anti-Luci**  **Anti-Raf-1**  **Anti-Atg12** | 1.4  3.5  3.2  1.2  3.9  2.9 | 0.1  0.05  0.1  0.05  0.9  0.2 | 0.25  0.001  0.01  0.5  0.05  0.01 | > 0.05  ≤ 0.01**  ≤ 0.01**  > 0.05  ≤ 0.05*  ≤ 0.01** |
|  |  |  |  |  |  |
